# Phonon dispersions and Fermi surfaces nesting explaining the variety of charge ordering in titanium-oxypnictides superconductors

**DOI:** 10.1038/srep29661

**Published:** 2016-07-19

**Authors:** Kousuke Nakano, Kenta Hongo, Ryo Maezono

**Affiliations:** 1 School of Information Science, JAIST, Asahidai 1-1, Nomi, Ishikawa 923-1292, Japan

## Abstract

There has been a puzzle between experiments and theoretical predictions on the charge ordering of layered titanium-oxypnictides superconductors. Unconventional mechanisms to explain this discrepancy have been argued so far, even affecting the understanding of superconductivity on the compound. We provide a new theoretical prediction, by which the discrepancy itself is resolved without any complicated unconventional explanation. Phonon dispersions and changes of nesting vectors in Fermi surfaces are clarified to lead to the variety of superlattice structures even for the common crystal structures when without CDW, including orthorhombic 2 × 2 × 1 one for BaTi_2_As_2_O, which has not yet been explained successfully so far, being different from tetragonal 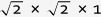 for BaTi_2_Sb_2_O and BaTi_2_Bi_2_O. The electronic structure analysis can naturally explain experimental observations about CDW including most latest ones without any cramped unconventional mechanisms.

Layered titanium oxypnictides, *A*Ti_2_*Pn*_2_O [*A* = Na_2_, Ba, (SrF)_2_, (SmO)_2_; *Pn* = As, Sb, Bi]^1^,^2^,^3^,^4^,^5^,^6^,^7^, have the common undistorted structure, as shown in Fig. 1, including Ti_2_O-plane that leads to quasi two-dimensional (2D) electronic structures. Yajima *et al*.^5^ synthesized BaTi_2_Sb_2_O and reported its superconductivity with the transition temperature, *T*_*c*_ = 1.2 K. Doan *et al*.^6^ also synthesized Ba_(1−*x*)_Na_*x*_Ti_2_Sb_2_O individually and reported its superconductivity with *T*_*c*_ = 5.5 K. Followed by their pioneering works, similar kinds of compounds, BaTi_2_Bi_2_O, BaTi_2_(Sb_1−*x*_Bi_*x*_)_2_O, BaTi_2_(Sb_1−*x*_Sn_*x*_)_2_O, Ba_1−*x*_K_*x*_Ti_2_Sb_2_O, and Ba_1−*x*_Rb_*x*_Ti_2_Sb_2_O, have been synthesized to get superconductivities, achieving the current highest *T*_*c*_ around 6.1 K^7^,^8^,^9^,^10^,^11^,^12^. Based on Allen-Dynes formalism^13^,^14^ within DFT, Subedi suggested a conventional BCS-type superconductivity mechanism holds in BaTi_2_Sb_2_O^15^. This theoretical finding was supported afterward by experiments of specific heats, NMR, and *μ*SR^16^,^17^,^18^, confirming full-gap BCS mechanism with *s*-wave paring for this compound. Although their *T*_*c*_ values themselves are relatively low compared with possibly conventional BCS-type mechanism, the superconductivity of BaTi_2_Sb_2_O and its relatives attracts special interests in the sense that their nominal electronic configurations, Ti^3 + ^(*d*^1^), are conjugate with those for cuprates superconductors^19^ with respect to the electron-hole symmetry. Quasi 2-dimensional (2D) transports in these systems also attract the common interest among those for cuprates^19^ as well as for iron arsenides superconductors^20^, leading to the arguments on the similarity of superconducting mechanisms^5^,^6^.

The possibilities of the density waves (DW) in these systems are one of the key concepts, which is common to low-dimensional transports possible for cuprates and iron arsenides. Anomalies in the temperature dependence of resistivity *ρ*(*T*) and magnetic susceptibility *χ*(*T*) at low temperature are sometimes observed in these systems, getting into an argument over if the anomalies can be attributed to the emergence of charge density waves (CDW) or spin density waves (SDW)^1^,^2^,^4^,^5^,^6^. Several DFT studies applied to BaTi_2_As_2_O and BaTi_2_Sb_2_O^21^,^22^,^23^ have reported the possibility of SDW, while it has not yet been observed experimentally, such as by NMR/NQR for Sb^121/123^ and *μ*SR for BaTi_2_As_2_O and BaTi_2_Sb_2_O^16^,^17^,^18^. Focusing on CDW, the nesting of Fermi surface matters, which is enhanced by 2D nature of Ti_2_O planes. While conventional models take a simple picture of 2D transport with Ti-3*d*_*xy*_ orbital only^5^, Singh^21^ clarified a more complicated 3D shape of Fermi surface in BaTi_2_Sb_2_O by taking into account several Ti-3*d* orbitals contributions. A similar shape was also predicted in BaTi_2_Bi_2_O, theoretically^24^. Indeed, such shapes have been recently observed by state-of-the-art Angle-Resolved Photo Emission Spectroscopy (ARPES) applied to BaTi_2_As_2_O and BaTi_2_Sb_2_O single crystals^25^,^26^. Subedi^15^ worked on BaTi_2_Sb_2_O using DFT and reported the possible lattice instability toward CDW with 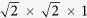 superstructure at low temperature. Experimentally, however, no such superlattice peaks were found by neutron and electron diffractions for this compound^17^,^27^, reviving the argument over if the anomalies in *ρ*(*T*) and *χ*(*T*) can be really attributed to the phonon-driven CDW or not. Though Frandsen *et al*.^27^ observed tiny lattice displacements from tetragonal to orthorhombic in both BaTi_2_As_2_O and BaTi_2_Sb_2_O, theoretically predicted 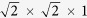 superlattice peaks^15^ could not be found experimentally. To account for this, they proposed a bit complicated picture that the intra-unit-cell nematic charge order, such as that observed in cuprates^28^,^29^, might be the origin of the anomalies in *ρ*(*T*) and *χ*(*T*).

Another important topic on this system is the ‘two-dome’ structure in the dependence of *T*_*c*_(*x*) on the concentration of ionic substitutions, *Pn* = Sb_*x*_Bi_(1−*x*)_ in BaTi_2_*Pn*_2_O^8^. Similar ‘two-dome’ structures are known also for cuprates^30^,^31^,^32^,^33^,^34^ and iron arsenides^35^,^36^ superconductors. The ‘two-dome’ structure can be regarded as a modification with a singularity put on the original single peak dependence. The singularity might be attributed to electron or spin orderings such as DW transition. Actually, a series of NMR experiments on LaFeAsO_1−*x*_H_*x*_^37^ identified the peak as being corresponding to the emergence of the new SDW phase related to the second *T*_*c*_ dome. This suggests that another origin of the second dome be different from that of the first dome. The ‘two-dome’ for BaTi_2_*Pn*_2_O, on the other hand, has not well been investigated so far.

As mentioned above, the layered titanium oxypnictides superconductors show a lot of similar phenomena to cuprates and iron-asenides superconductors. The investigation of DW transition in these oxypnictides could then be one of the most important clues to understanding of the physics of high-*T*_*c*_ superconducting mechanism. In the present study, we investigated the possibility of DW transition in BaTi_2_*Pn*_2_O (*Pn* = As, Sb, Bi) using DFT-based phonon analysis. Because of the common lattice structure when without DW, we could perform systematic and careful comparisons among the three compounds, putting the same computational conditions to suppress artifacts as less as possible. As a result, we found a new possibility of orthorhombic 2 × 2 × 1 superlattice structure for BaTi_2_As_2_O, which is different from the previous prediction by Subedi^15^ for BaTi_2_Sb_2_O, tetragonal 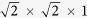. Our theoretical finding can provide a more natural explanation for the structural transition and the weak superlattice peaks observed by Frandsen *et al*.^27^ in terms of the conventional phonon-driven CDW, not by such a complicated mechanism as intra-unit-cell nematic charge ordering, as given in their papers^28^,^29^. The finding might also account for the anomalies of *ρ*(*T*) and *χ*(*T*) being attributed to the lattice instability. While BaTi_2_Bi_2_O does not show anomalies in *ρ*(*T*) and *χ*(*T*), we found lattice instability possibly inducing a tetragonal 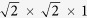 superlattice structure. Such a discrepancy is observed in a LaO_0.5_F_0.5_BiS_2_ superconductor as well^38^. To account for this, Yildirim^39^ argued the possibility of an unconventional superconducting mechanism in which the inherent lattice instability plays an important role in the Cooper paring. The similarity of the discrepancy for the present case might imply the similar unconventional mechanism for BaTi_2_Bi_2_O.

## Results

### Lattice instabilities from undistorted structures

**Figure 2:**
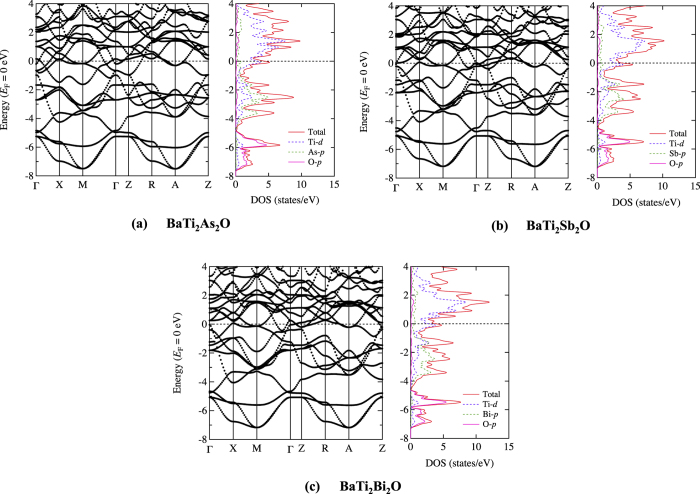
Electronic band structures and densities of states for BaTi_2_*Pn*_2_O (*Pn* = (**a**) As, (**b**) Sb, and (**c**) Bi) under *P*4/*mmm* symmetry. Each Fermi energy is defined as zero.

**Figure 3:**
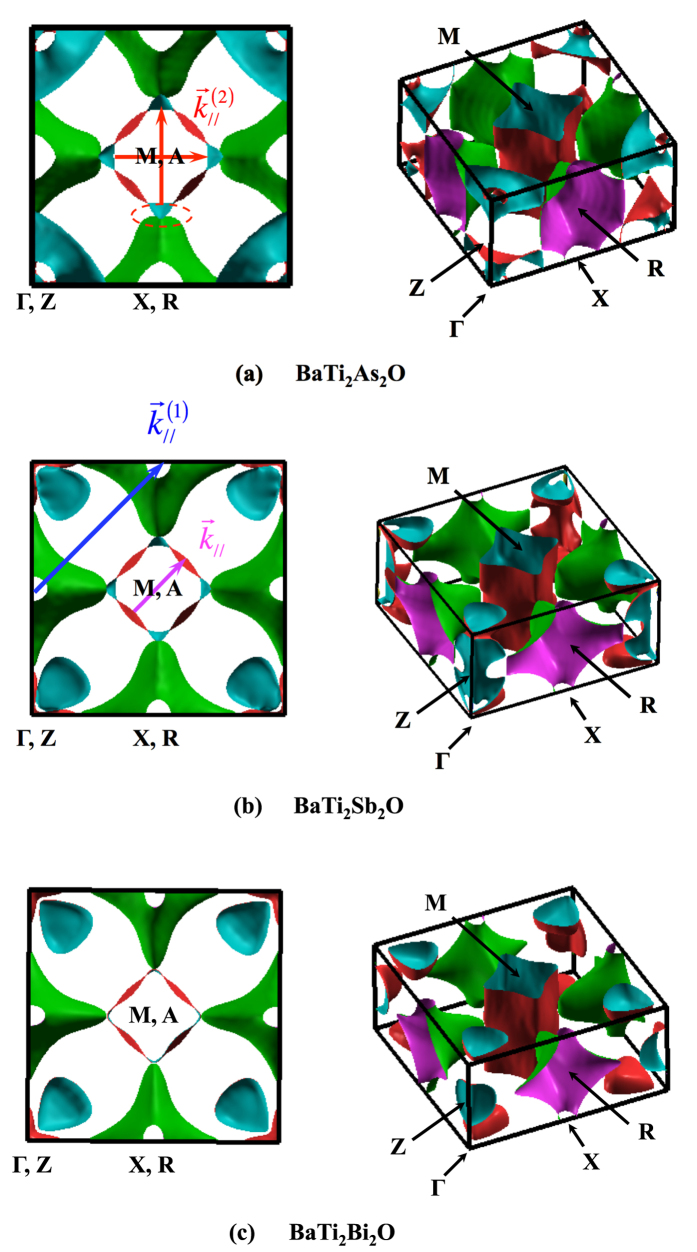
Fermi surfaces of BaTi_2_*Pn*_2_O under *P*4/*mmm* symmetry (*Pn* = (**a**) As, (**b**) Sb, and (**c**) Bi). Possible nesting vectors are also depicted (see text for notation). Note that Γ point is located at the corner, as shown in the figure.

Electronic band structures and densities of states (DOS) for undistorted structures are shown in Fig. 2. Figure 3 highlights the corresponding Fermi surfaces with possible nesting vectors. Overall features agree well with previous DFT results by Yu *et al*.^23^, Singh^21^ and Suetin *et al*.^24^ for each compound, justifying no specific artifacts due to any choices of computational conditions. Note that our optimized geometry parameters are also in good agreements with the previous calculations (See Supplementary Note 1).

**Figure 4:**
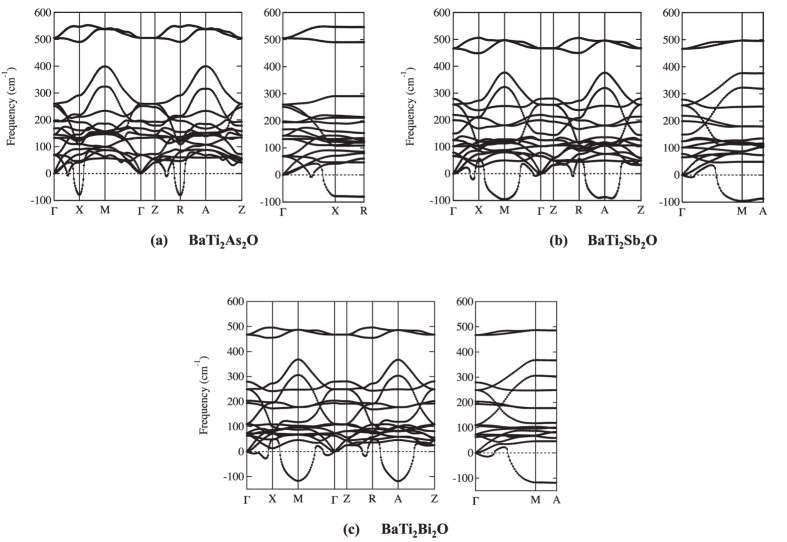
Phonon dispersions of BaTi_2_*Pn*_2_O under *P*4/*mmm* symmetry (*Pn* = (**a**) As, (**b**) Sb, and (**c**) Bi).

**Figure 5:**
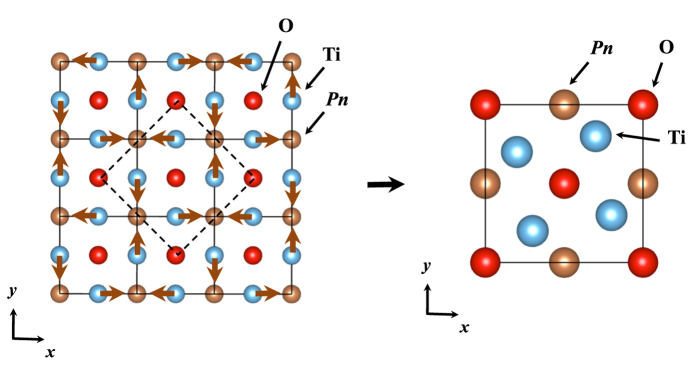
Atomic displacements corresponding to the negative (imaginary) phonon mode at *M* (1/2, 1/2, 0) point, leading to 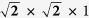 superlattice for BaTi_2_*Pn*_2_O (*Pn* = Sb and Bi). Dashed lines in the left panel stand for the unit cell of the superlattice, drawn in the right panel again with displaced atomic positions after the rotation by 45 degree. The symmetry of the superlattice is *P*4/*mbm* (No. 127).

Phonon dispersions for undistorted structures are shown in Fig. 4. Despite the common crystal structure, we see that the phonon instabilities appear around different points, *M* and *A* for *Pn* = Sb, Bi, while *X* and *R* for *Pn* = As [hereafter, all the 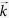 and 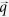 points are labeled according to Brillouin-zone database on the *Bilbao Crystallographic Server*^40^]. The former instabilities (for *Pn* = Sb, Bi) are consistent with the previous calculations by Subedi^15^ on *Pn* = Sb. The latter instability (for *Pn* = As), on the other hand, has been never reported before, so we carefully examined to confirm that the result does not depend on the choice of pseudo potentials. For *Pn* = Sb and Bi, the instability occurs around *M* and *A*, corresponding to (*q*_*x*_, *q*_*y*_) = (1/2, 1/2) [hereafter a unit of 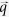 is 2*π*/*a*.]. For *q*_*z*_ direction, there is no specific dependence, as shown in the dispersion along *M* to *A* in the right panel of Fig. 4(b,c). From phonon pDOS (partical DOS), we can identify which vibration modes lead to the instability toward the superlattice. It is found from phonon pDOS that the negative (imaginary) frequencies mainly come from Ti ‘in-plane’ (within *xy* plane) vibrations (See Supplementary Note 2). We therefore concentrate on the representative case with *M*, (*q*_*x*_, *q*_*y*_, *q*_*z*_) = (1/2, 1/2, 0), corresponding to 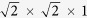 superlattice structure. By analyzing the dynamical matrices, we can further identify the superlattice structure shown in Fig. 5. Note that this is the same structure as that predicted previously by Subedi^15^ for *Pn* = Sb.

**Figure 6:**
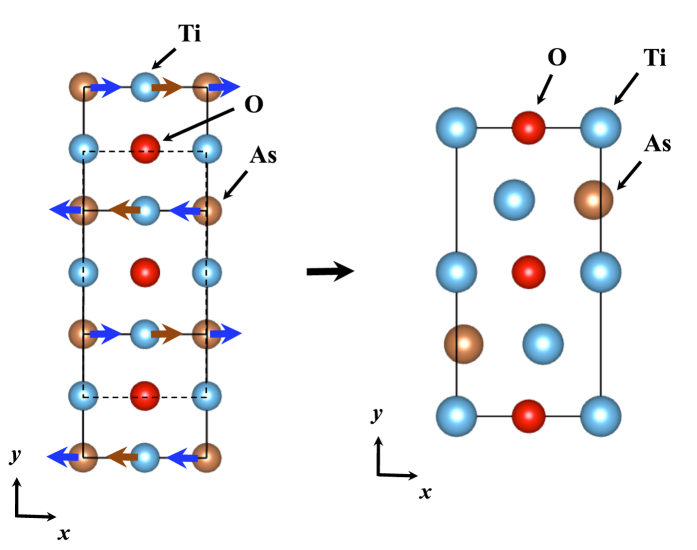
Atomic displacements corresponding to the negative (imaginary) phonon mode at *X* (0, 1/2, 0) point, leading to 1 × 2 × 1 superlattice for BaTi_2_As_2_O. Dashed lines in the left panel stand for the unit cell of the superlattice, drawn in the right panel again with displaced atomic positions. The symmetry of the superlattice is *Pbmm* (No. 51).

The same scheme is applied to *Pn* = As with the instability around *X* and *R*, corresponding to (*q*_*x*_, *q*_*y*_) = (0, 1/2): For *q*_*z*_ direction, there is no specific dependence, as shown in the dispersion along *X* to *R* in the right panel of Fig. 4(a). From phonon pDOS, we can identify that the vibrations for the instability come from Ti and As ‘in-plane’ vibrations (See Supplementary Note 2). We therefore take a representative mode at *X*, (*q*_*x*_, *q*_*y*_, *q*_*z*_) = (0, 1/2, 0). By analyzing the dynamical matrices, we get the superlattice structure shown in Fig. 6, 1 × 2 × 1 superlattice.

### Superlattice structures and *T*_*c*_

**Figure 7:**
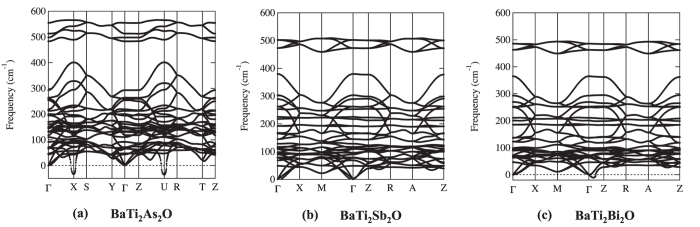
Phonon dispersions of superlattice structure for BaTi_2_*Pn*_2_O (*Pn* = (**a**) As-1 × 2 × 1, (**b**) Sb-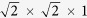, and (**c**) Bi-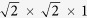.

**Figure 8:**
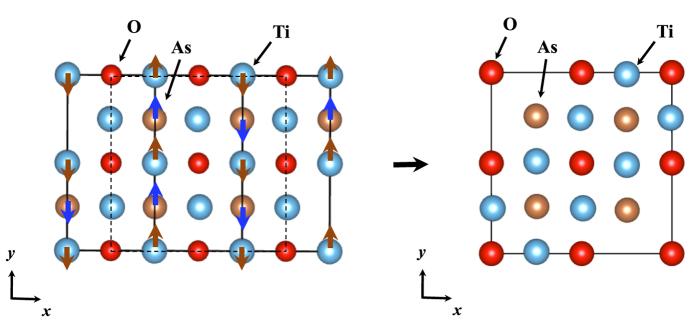
Atomic displacements for 1 × 2 × 1 BaTi_2_As_2_O corresponding to the negative (imaginary) phonon mode at *X* (1/2, 0, 0) point, leading to orthorhombic 2 × 2 × 1 superlattice. Dashed lines in the left panel stand for the unit cell of the superlattice, drawn in the right panel again with displaced atomic positions. The symmetry of the 2 × 2 × 1 superlattice is *Pbam* (No. 55).

The negative modes appearing in Fig. 4 are expected to disappear when we further relax the lattices along the negative modes to get superlattices. Resultant phonon dispersions are shown in Fig. 7. For 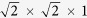 superlattices of Sb and Bi, the negative modes have disappeared assuring the superlattice as the final stable structures [the negative mode seen around Γ for Bi is due to the well-known artifact coming from the discreteness of Fast Fourier Transform (FFT) grid]. For As, on the other hand, the negative modes at *X* and *U* point still remain. Taking the representative *X* point, this implies the further superlattice transition toward 2 × 2 × 1. The phonon pDOS analysis shows that the superlattice deformation still comes from ‘in-plane’ vibrations of Ti and As, actually leading to the superlattice structure as shown in Fig. 8. The geometry optimization along this deformation gives lattice parameters, *a* = 8.122 Å, *b* = 8.108 Å, and *c* = 7.401 Å (See Supplementary Note 4). The orthorhombicity parameter is *η* = 2 × (*a* − *b*)/(*a* + *b*) = 0.171%. To finalize the verification, we ought to examine if the negative modes surely disappear in the 2 × 2 × 1 superlattice. It was, however, intractable because four times enlarged unit cell requires 4^3^ times more computational cost and lower symmetry makes more demands for *k*- and *q*-mesh samplings over larger reciprocal space.

Superconducting transition temperatures, *T*_*c*_, are estimated using Allen-Dynes formula^14^, as shown in Table VI in Supplementary Information. The parameters used in the formula are also tabulated. Even with imaginary frequencies, *T*_*c*_ can be estimated just by ignoring the contributions^15^, being the case for 1 × 1 × 1. For 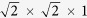 where all the negative modes disappear, the estimation gets to be more plausible. There seems, however, little change in the estimation from that in 1 × 1 × 1. The present estimations, *T*_*c*_ = 2.30 (2.45) K for *Pn* = Sb (Bi), are consistent with experiments reporting *T*_*c*_ = 1.2 (4.6) K for *Pn* = Sb (Bi)^5^,^7^, as well as that by previous DFT study, *T*_*c*_ = 2.7 K for *Pn* = Sb^15^. For 1 × 2 × 1 (*Pn* = As), the estimation was made still under the existence of negative modes, and the more plausible estimation for 2 × 2 × 1 was intractable as mentioned above. The estimations, *T*_*c*_ = 6.93 K (1 × 1 × 1) and 8.31 K (1 × 2 × 1) seem incompatible with experiments of BaTi_2_As_2_O exhibiting no superconductivity so far^4^,^8^. We might expect much lower *T*_*c*_ obtained for the 2 × 2 × 1 superlattice structure.

## Discussions

### Comparison with experiments

The orthorhombic 2 × 2 × 1 superlattice structure, obtained here for BaTi_2_As_2_O, would attract interests in connection with experimental observations. Frandsen *et al*. actually observed a lattice structural transition from tetragonal *P*4/*mmm* to orthorhombic *Pmmm* by neutron diffractions. They also reported very weak superlattice peaks corresponding to *q*_*Hx*_ = (1/2, 0, 0) or *q*_*Hy*_ = (0, 1/2, 0), which disappears at higher temperatures, by electron diffractions^27^. Since BaTi_2_As_2_O has the same *P*4/*mmm* parent structure as BaTi_2_Sb_2_O, they might expect a tetragonal 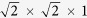 superlattice according to the previous DFT prediction by Subedi^15^ for *Pn* = Sb. The lack of the expected 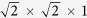 superlattice and the observation of the structural transition were explained by rather complicated mechanism such as intra-unit-cell nematic charge ordering, as an analogue of that in cupurates^28^,^29^. The observed weak peaks of *q*_*Hx*_ or *q*_*Hy*_ was then regarded as being non-intrinsic, attributed to poly-crystalline grain boundary effects. Our finding of orthorhombic 2 × 2 × 1 superlattice here can instead account for the observations, as intrinsic, more naturally in terms of the lattice instability due to conventional phonon-driven CDW. Note that our optimized geometry gives the orthorhombicity parameter, *η* = 2 × (*a* − *b*)/(*a* + *b*) = 0.171%, which is comparable with the experimental value *η* = 0.22%^27^. Though *q*_*Hx*_ or *q*_*Hy*_ is naturally explained, the present result also leads to the emergence of *q*_*Hxy*_ = (1/2, 1/2, 0), which is not explicitly reported in the work by Frandsen *et al*.^27^ Looking at their TEM photo in the paper^27^, it is actually quite difficult to distinguish the *q*_*Hxy*_ peak from much brighter spots in the immediate vicinity. Polycrystalline sample qualities and weak intensities of the peak might also matter. We expect that further careful investigation would find *q*_*Hxy*_ peak corresponding to the present 2 × 2 × 1 superlattice structure.

Unlike *Pn* = As, the other two compounds are predicted to have 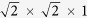 superlattices in our calculations, which is consistent with the preceding work by Subedi^15^ for *Pn* = Sb. Then a question arises asking why only *Pn* = As takes the different superlattice structure. This can be explained by the nesting of Fermi surfaces, shown in Fig. 3. Because of the cylinderical shape, every compound has a nesting vector 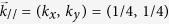 [hereafter a unit of 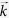 is 2*π*/*a*]. around *M* and *A* points, as previously pointed out by Yu *et al*.^23^ for *Pn* = As . Another possible nesting around *X* and *R* is described by the vector 
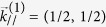
, corresponding to the negative phonons we get around *M* and *A* in phonon Brillouin zones for *Pn* = Sb, Bi. This nesting has already been pointed out by Singh^21^ and Subedi^15^ for *Pn* = Sb. Looking carefully at the Fermi surface of *Pn* = As, we see the flattening of the cusp around *X* and *R* points (as shown by a dashed oval in Fig. 3(a)), leading to new nesting vectors, 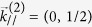 and (1/2, 0). They correspond to the negative phonons around *X* and *R* appearing only for *Pn* = As. We note that 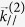 have already been mentioned by Yu *et al*.^23^ for *Pn* = As, but its relation to the phonon instability has not been discussed so far. Possible reasons why the flattening occurs only for As are discussed in the next section. Since all the above stories can be made only within the electronic Fermi surfaces, one might consider the phonon evaluations not necessarily required. We note, however, that there are several 2D chalcogenide systems where CDW cannot be explained only by the electronic Fermi surfaces, but accounted for when the phonon dispersions are evaluated^41^,^42^,^43^,^44^,^45^. We discuss this in the later section. It is further interesting if CDW superlattice transitions predicted here could be related to the anomalies of *ρ*(*T*) and *χ*(*T*) at low temperature.

The 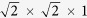 superlattice structure for BaTi_2_Sb_2_O is predicted not only by the present work but also by Subedi^15^. However, such a superlattice has not yet been observed experimentally so far by any diffraction experiments such as neutron and electron diffractions^17^,^27^. Frandsen *et al*. observed a subtle structural distortion from *P*4/*mmm* to *Pmmm* by neutron diffraction measurement at low temperature^27^, which is not consistent with the prediction. Note that all the above diffraction experiments are applied to polycrystalline samples. Interestingly, Song *et al*. has very recently reported the existence of (1/2, 1/2) nesting vector by ARPES and STM measurements applied to high-quality single crystals, being consistent with the theoretical predictions^26^. The superlattice structure is hence expected to be detected by further diffraction measurements. The theoretically estimated distortion here is quite small, 0.14 Å (See Supplementary Note 4), being in agreement with the previous calculation by Subedi^15^, so careful detections would be required for experiments. As described in the previous section, the estimated *T*_*c*_ is almost consistent with experiments, supporting that the compound is a conventional BCS-type superconductor, being consistent with the previous conclusion by Subedi^15^.

In contrast to the other two compounds, there are little experiments on BaTi_2_Bi_2_O because of the difficulty of sample preparation mainly due to the significant instability under the air and moisture^7^. As far as we have known, there is no previous research on phonon dispersions on this system. The anomalies of *ρ*(*T*) and *χ*(*T*) disappear with increasing *T*_*c*_ when Sb is gradually substituted by Bi, as reported experimentally^7^. Though there is no direct evidence by diffraction experiments, it seems, then, the present consensus on this compound that there is no instability toward CDW, which would be contradicting to our result here. The spin polarization, not taken into account here, may be one of the possibilities to modify the nesting, for instance via the spin-orbit coupling, accounting for this discrepancy, but it is reported, at least for DOS, the effect matters little^21^. A similar discrepancy between negative phonon predictions^39^,^46^ and unobserved structural instability is known for a superconductor, LaO_0.5_F_0.5_BiS_2_^38^. A large phonon instability toward a static CDW was estimated theoretically^39^, while no anomaly in *ρ*(*T*) and *χ*(*T*) has been observed experimentally^38^,^47^. Yildirim^39^ then argued the possibility of an unconventional superconducting mechanism in which inherent lattice instabilities have an important role on the Cooper paring in this compound. A more recent neutron diffraction experiment^48^ reported that the local distortion of the atomic position of S around Bi is detected under *T*_*c*_, attracting an attention in connection with the unconventional mechanism. In our case of BaTi_2_Bi_2_O, the stabilization energy is evaluated around 23.3 meV/UnitCell, being much larger than that of LaO_0.5_F_0.5_BiS_2_ (~10 meV/UnitCell)^39^. In terms of the magnitude of the displacement, it is 0.16 Å for BaTi_2_Bi_2_O (See Supplementary Note 4), which is the same as 0.16 Å for LaO_0.5_F_0.5_BiS_2_^39^. In addition, the 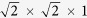 superlattice structure obtained by analyzing dynamical matrices for BaTi_2_Bi_2_O does not show any negative phonon frequency (Fig. 7(c)). Therefore, the lattice instability is also expected to be static. The similarity might imply the similar unconventional mechanism also for BaTi_2_Bi_2_O. If it were so, the substitution of Sb by Bi would introduce the unconventionality to the conventional BCS of BaTi_2_Sb_2_O^16^,^17^,^18^. The introduction might account for the two-dome structure appeared by the substitution^8^.

### Possible mechanism for variety of superlattices

A natural question consequently arising would be asking why the new nesting vectors 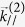 appear only for *Pn* = As. The vectors are caused by the flattening of the ‘nose’ of Fermi surfaces directing toward the central cylinder from four equivalent outsides. Interestingly, the similar flattened ‘nose’ was actually reported in the paper by Singh^21^ (in its Fig. 7), shown as the ‘Fermi surfaces’ below *E*_*F*_ by 0.1 eV for *Pn* = Sb. Looking at our Fig. 2, we observe that the Fermi level seems to be approaching down toward the DOS peak as *Pn* changes from Bi and Sb to As. This can be regarded as if *E*_*F*_ effectively behaves like the ‘sea-level down’ *Pn* = As with a fixed landscape of *Pn* = Sb. The Fermi surface of *Pn* = As would therefore correspond to that of *Pn* = Sb with negative energy shift, as shown in ‘Fig. 7 by Singh’^21^. The ‘fixed landscape’, namely the ‘rigid band picture’ near to Fermi level, can be justified to some extent because they are mainly composed of Ti-*d* orbital contributions as shown in Fig. 2. The reason why the ‘sea-level’ gets down when *Pn* is substituted into As can roughly be accounted as follows: As a rough estimation of how *Pn* affects to shift *E*_*F*_, we can start with its ‘HOMO’ level of the isolated atom [HOMO stands for ‘Highest Occupied Molecular Orbital’ though the orbital in the present context is not molecular but isolated atomic orbital. We use ‘HOMO’ rather than HOAO just because the latter is not so commonly spread abbreviation. We expect this doesn’t matter so much even it is used for isolated atom.]. Namely, a negatively deeper ‘HOMO’ would contribute to attract Ti-electrons more strongly and make *E*_*F*_ lower. Noticing the deeper ‘HOMO’ corresponds to the larger ionic potential, we expect that the lighter element (As) has deeper ‘HOMO’ because the potential is enhanced by the less screening of the nucleus attractions by fewer inner electrons^49^. The deeper ‘HOMO’ also corresponds to the larger electronegativity, which is actually 2.18 for As while 2.05 (2.02) for Sb (Bi) by Pauling scale. Similar negativities for Sb and Bi can account for the common nesting vectors of these compounds, being different from that of As. Summarizing the above, the negatively deeper ‘HOMO’ level of As can attract Ti-electrons more strongly and effectively push *E*_*F*_ down when it forms pnictides, and then the Fermi surface changes to get flattened ‘nose’ as depicted in ‘Fig. 7 of Singh’^21^.

Though we could not make clear explanations here, we must note that the nesting vector cannot solely account for the superlattice instabilities even in the present case. In addition to 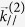 for *Pn* = As, 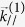 and 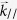 may be regarded as possible nesting vectors. The Kohn anomalies corresponding to 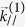 and 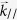 are, however, not present in the phonon dispersion. This fact might be related to recent intensive discussions about the Kohn anomaly^50^: Some studies^41^,^42^,^43^,^44^,^45^ insist that the imaginary phonons in quasi 2D systems are dominated not mainly by the nesting of electronic structures but rather primarily by the wave vector dependence of the electron-phonon coupling, 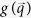. There exists, however, such quasi 2D systems^51^ where their superlattice instabilities can clearly be explained by the nesting vectors. To investigate if our system corresponds to which case or that lying in between, it is quite intriguing to analyze 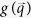 for *Pn* = As, but unfortunately we cannot perform any of such phonon calculations under the perfect disappearance of imaginary frequencies because of too costly calculations for the 2 × 2 × 1 superlattice.

## Methods

All the calculations were done within DFT using GGA-PBE exchange-correlation functionals^52^, implemented in Quantum Espresso package^53^. After carefully examining the artifacts due to the choice of pseudo potentials (PP), we provide here the final results mainly obtained by the PAW^54^ framework of the valence/core separation of electrons. The implementation of PAW adopted here takes into account the relativistic effects within the extent of the scalar-relativistic theory upon a careful comparison with all-electron calculations by Wien2k^55^. We restricted ourselves to spin unpolarized calculations, anticipating that the spin polarization affects little as supported by several experiments^16^,^17^,^18^. Lattice instabilities are detected by the negative (imaginary) phonon dispersions evaluated for undistorted lattice structures. Taking each of the negative phonon modes, the structural relaxations along the mode are evaluated by the BFGS optimization scheme with the structural symmetries fixed to *Pbmm* (1 × 2 × 1) and *Pbam* (2 × 2 × 1) for BaTi_2_As_2_O, *P*4/*mbm* (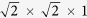) for BaTi_2_Sb_2_O and BaTi_2_Bi_2_O. For phonon calculations, we used the linear response theory implemented in Quantum Espresso package^56^. Crystal structures and Fermi surfaces are depicted by using VESTA^57^ and XCrySDen^58^, respectively.

To deal with the three compounds systematically, we took the same conditions for plane-wave cutoff energies (*E*_cut_), *k*-meshes, and smearing parameters. The most strict condition among the compounds is taken to achieve the convergence within ±1.0 mRy in the ground state energies of undistorted (superlattice) systems, resulting in 
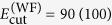
 Ry for wavefunction and 
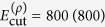
 Ry for charge densities. For *T*_*c*_ evaluation, we adopted unshifted *k*-meshes centered at Γ-point. Denser *k*-meshes should be taken for electron-phonon calculations because of the double-delta integrations^59^. For undistorted systems, (8 × 8 × 4) *k*-meshes were used for the Brillouin-zone integration. Phonon dispersions were calculated on (8 × 8 × 4) *q*-meshes. Denser *k*-meshes, (24 × 24 × 12), were used for the double-delta integrations in electron-phonon calculations. For distorted BaTi_2_As_2_O superlattices, (8 × 4 × 4) and (4 × 4 × 4) *k*-meshes were used for 1 × 2 × 1 and 2 × 2 × 1 superlattices, respectively. Phonon dispersions were calculated on (8 × 4 × 4) and (4 × 4 × 4) *q*-meshes. Denser *k*-meshes, (24 × 12 × 12), were used for the double-delta integrations in electron-phonon calculations of the 1 × 2 × 1 superlattice. For distorted BaTi_2_Sb_2_O and BaTi_2_Bi_2_O superlattices, (6 × 6 × 6) *k*-meshes are used. Phonon dispersions were calculated on (6 × 6 × 6) *q*-meshes. Denser *k*-meshes, (18 × 18 × 18), were used for the double-delta integrations in electron-phonon calculations. The Marzari-Vanderbilt cold smearing scheme^60^ with a broadening width of 0.01 Ry was applied to all the compounds. To estimate *T*_*c*_, we used Allen-Dynes formula^13^,^14^ implemented in Quantum Espresso^53^, with the effective Coulomb interaction μ*, being chosen 0.1 empirically (See Supplementary Note 3).

## Additional Information

**How to cite this article**: Nakano, K. *et al*. Phonon dispersions and Fermi surfaces nesting explaining the variety of charge ordering in titanium-oxypnictides superconductors. *Sci. Rep*. **6**, 29661; doi: 10.1038/srep29661 (2016).

## Supplementary Material

Supplementary Information

## Figures and Tables

**Figure 1:**
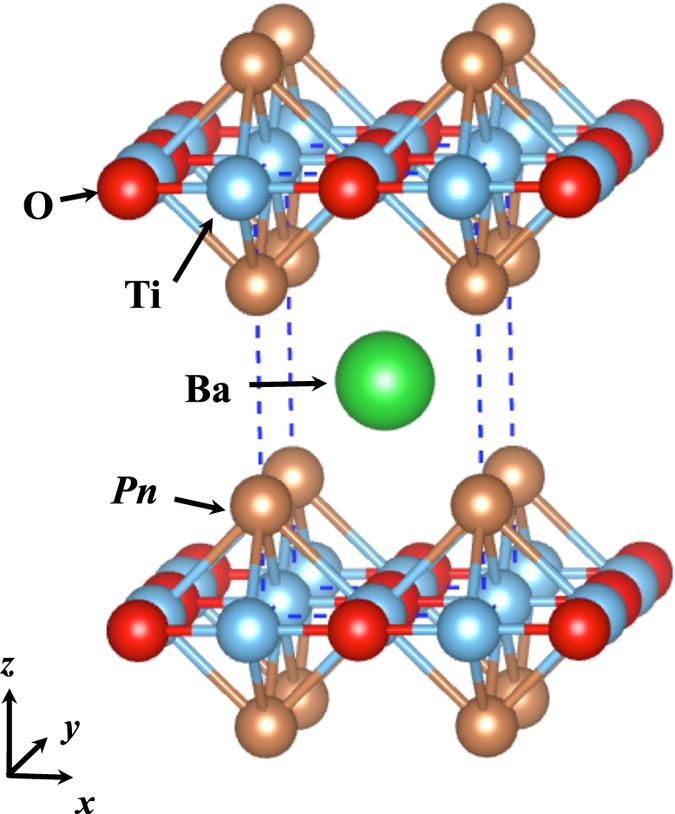
Undistorted crystal structures of BaTi_2_*Pn*_2_O (*Pn* = As, Sb, and Bi). The crystal symmetry is *P*4/*mmm* (No. 123).
