# Supplementary material for: Phonon dispersions and Fermi surfaces nesting explaining the variety of charge ordering in titanium-oxypnictides superconductors
Source: Sci Rep. 2016 Jul 19;6:29661. doi: 10.1038/srep29661 (PMC4949430; doi:10.1038/srep29661)
Supplement: Supplementary Information [file srep29661-s1.pdf]

Supplementary information for phonon dispersions and Fermi surfaces nesting explaining the variety of charge ordering in titanium-oxypnictides superconductors

Kousuke Nakano[1], Kenta Hongo[1], and Ryo Maezono[1]
[1] *School of Information Science, JAIST, Asahidai 1-1, Nomi, Ishikawa 923-1292, Japan. Correspondence and requests for materials should be addressed to K.N. (email:kousuke_1123@icloud.com) or R.M. (email:rmaezono@mac.com)*
(Dated: June 6, 2016)

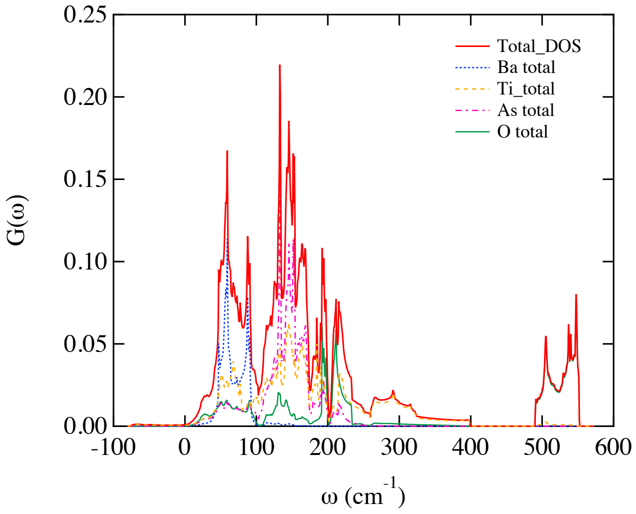

(a) $BaTi_2As_2O$

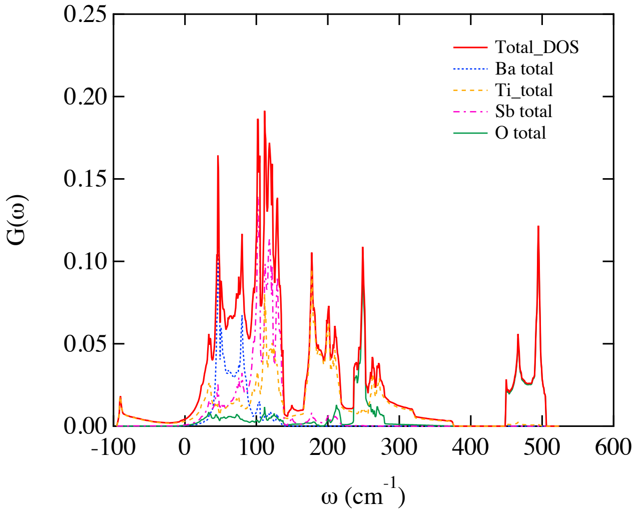

(b) $BaTi_2Sb_2O$

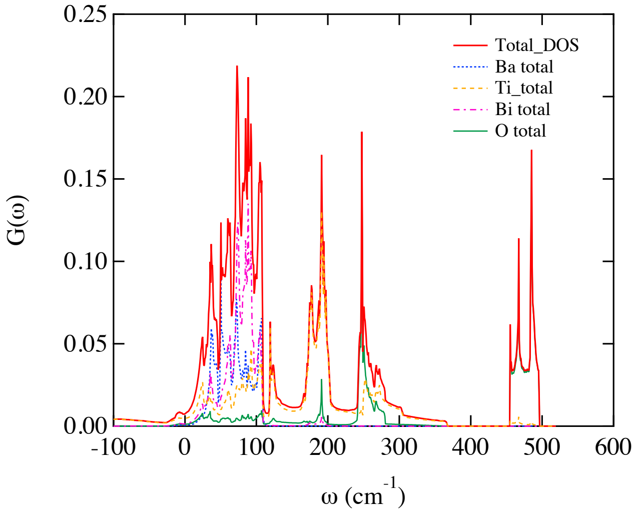

(c) $BaTi_2Bi_2O$

FIG. 1. Total and partial phonon density of states of $BaTi_2Pn_2O$ under $P4/mmm$ symmetry (Pn = (a) As, (b) Sb, and (c) Bi).

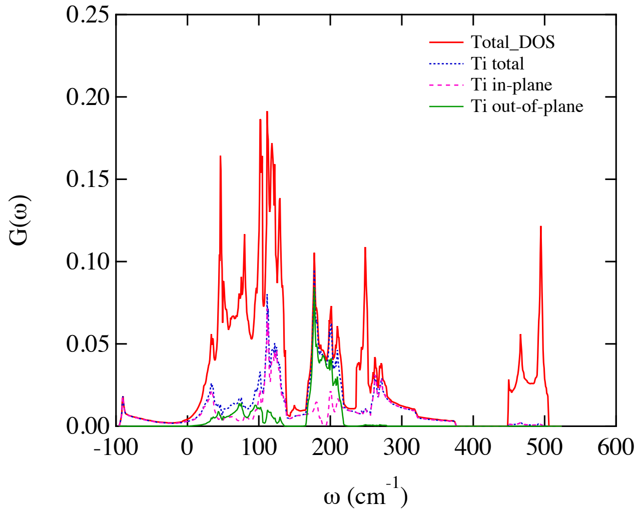

(a) $BaTi_2Sb_2O$

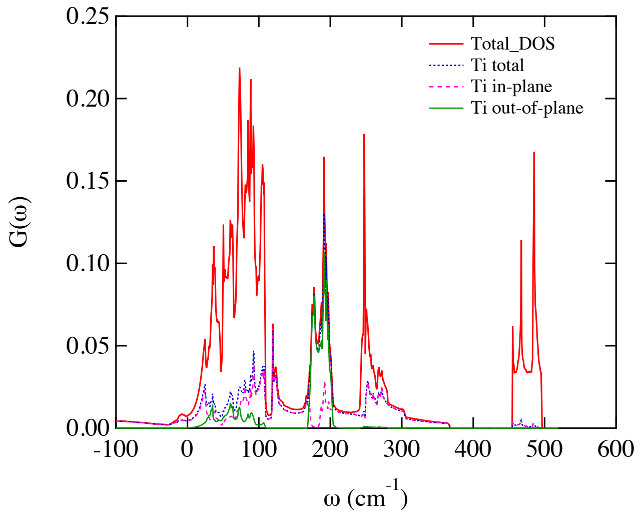

(b) $BaTi_2Bi_2O$

FIG. 2. Partial density of states for phonons in $BaTi_2Pn_2O$ (Pn = Sb and Bi), divided into the contributions from in-plane and out-of-plane vibrations of Ti atoms.

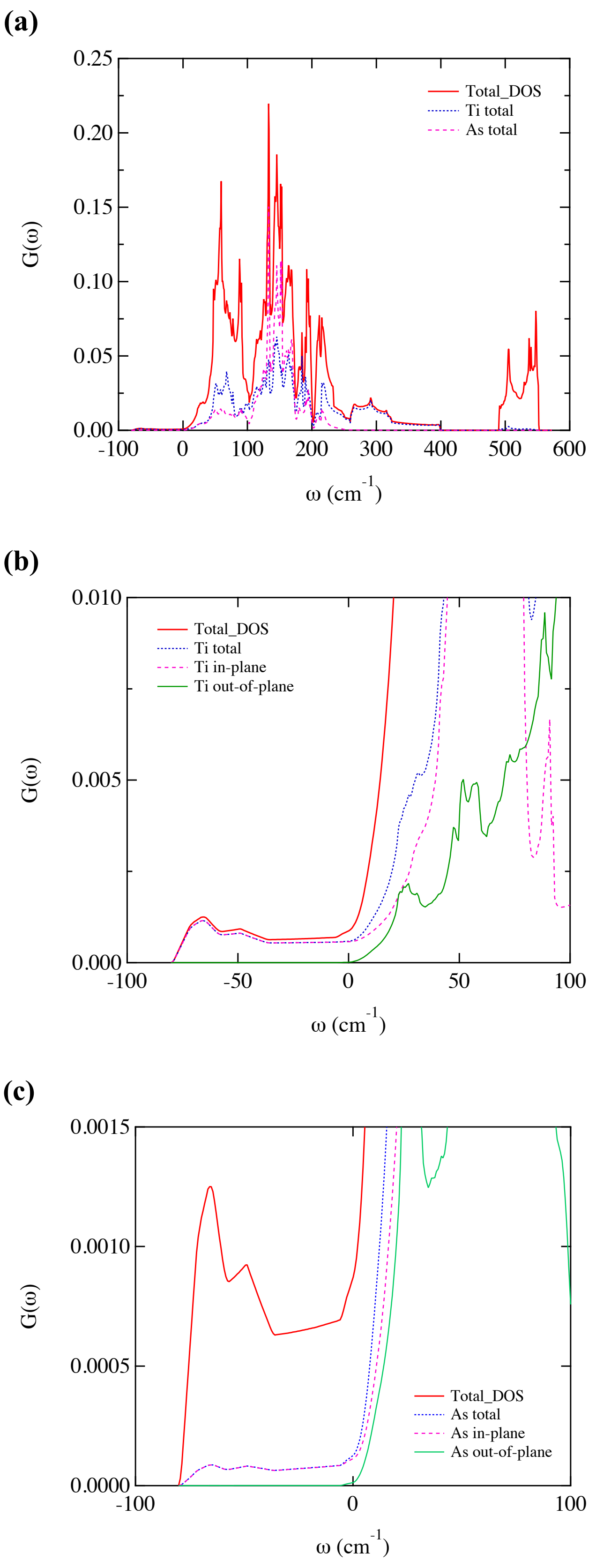

FIG. 3. Partial density of states for phonons in $BaTi_2As_2O$, divided into the contributions from in-plane and out-of-plane vibrations of Ti and As atoms.

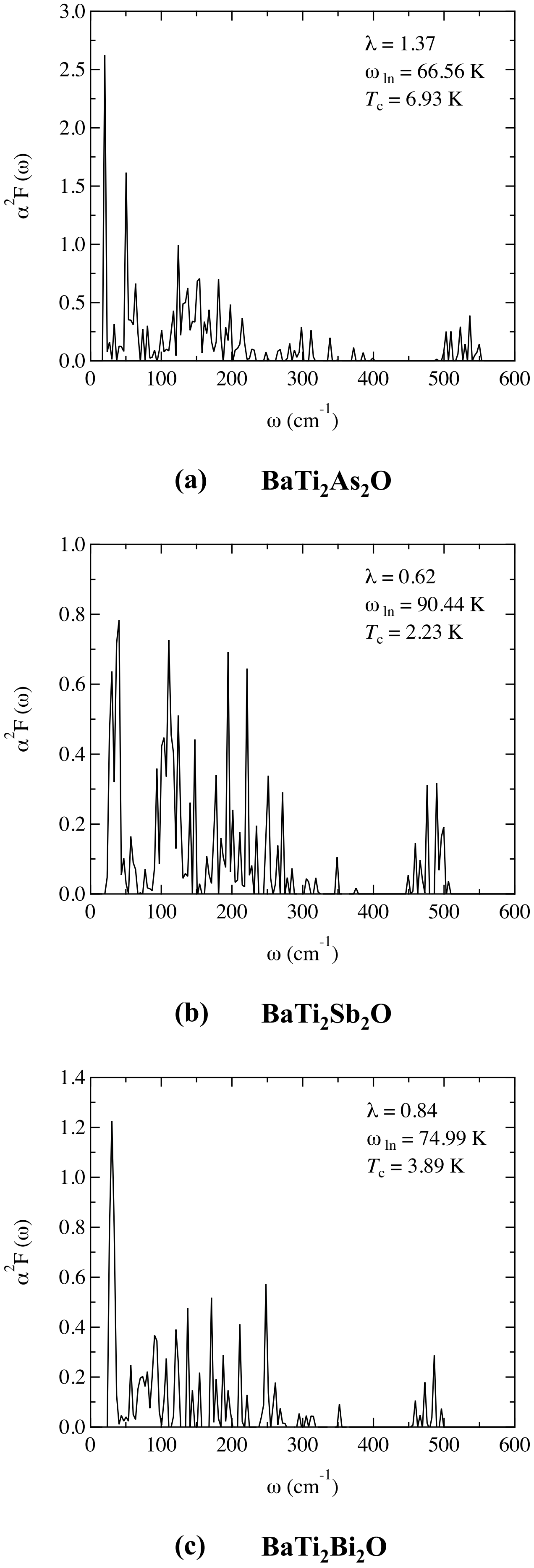

FIG. 4. Eliashberg spectral function $\alpha^2F(\omega)$ for (a) $BaTi_2As_2O$, (b) $BaTi_2Sb_2O$ and (c) $BaTi_2Bi_2O$ under $P4/mmm$ symmetry. The imaginary frequencies are not taken into account.

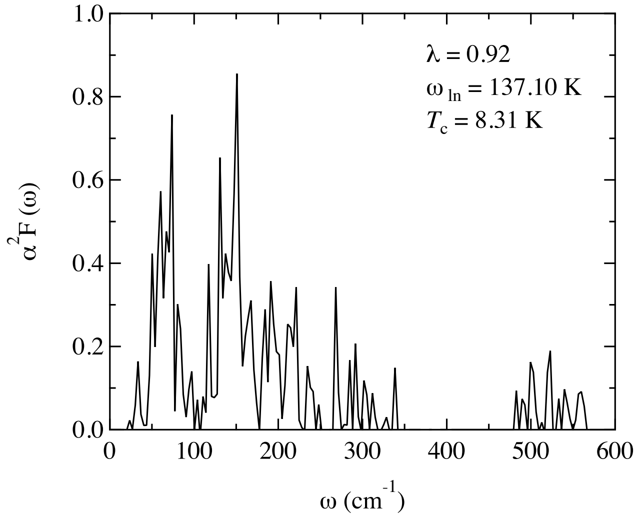

(a) $BaTi_2As_2O$

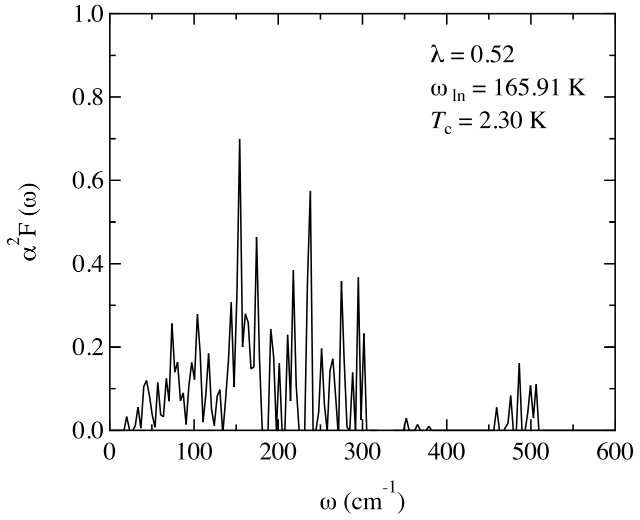

(b) $BaTi_2Sb_2O$

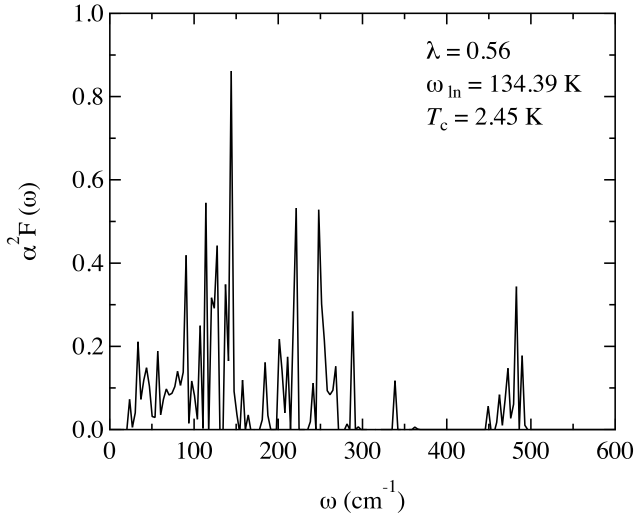

(c) $BaTi_2Bi_2O$

FIG. 5. Eliashberg spectral function $\alpha^2F(\omega)$ for superlattice structures, (a) $BaTi_2As_2O$-$1 \times 2 \times 1$, (b) $BaTi_2Sb_2O$-$\sqrt{2} \times \sqrt{2} \times 1$ and (c) $BaTi_2Bi_2O$-$\sqrt{2} \times \sqrt{2} \times 1$. The imaginary frequencies are not taken into account for $BaTi_2As_2O$.

TABLE I. Optimized lattice constants, a and c, and z-components of Pn atomic positions of $BaTi_2Pn_2O$ (Pn = As, Sb, Bi) under $P4/mmm$ symmetry, compared with experiments and other DFT results. All units are given in Å.

	Experiments			GGA-PBE (present)			GGA-PBE (Wien2k)		
Compounds	a	c	Pn z-pos.	a	c	Pn z-pos.	a	c	Pn z-pos.
$BaTi_2As_2O$	4.046[1]	7.272[1]	0.2440[1]	4.058	7.393	0.2422	4.057[2]	7.263[2]	0.2427[2]
$BaTi_2Sb_2O$	4.110[3]	8.086[3]	0.2487[3]	4.089	8.285	0.2451	4.116[2]	8.107[2]	0.2467[2]
$BaTi_2Bi_2O$	4.123[4]	8.345[4]	0.2513[4]	4.118	8.630	0.2481	4.122[5]	8.547[5]	0.2523[5]

TABLE II. Internal atomic positions of $BaTi_2As_2O$ superlattice structure ($1 \times 2 \times 1$, No.51 $Pbmm$), given in factional coordinates for the lattice constants, a = 4.060 Å, b = 8.110 Å, and c = 7.401 Å. All the atomic positions and lattice constants are simultaneously optimized by GGA-PBE. The magnitude of distortions in terms of the orthogonal index is evaluated as, $\eta = 2\times(a-1/2b)/(a+1/2b) = 0.115\%$, being fairly coincidence with the experimental value from neutron diffraction, [6] $\eta = 0.22\%$.

Atom	Site	x	y	z
Ba	$2d$	0.5000	0.5000	0.5000
Ti	$2a$	0.0000	0.0000	0.0000
Ti	$2e$	0.4851	0.2500	0.0000
As	$4k$	0.0040	0.2500	0.2422
O	$2c$	0.5000	0.0000	0.0000

TABLE III. Internal atomic positions of $BaTi_2Sb_2O$ superlattice structure ($\sqrt{2} \times \sqrt{2} \times 1$, No.127 $P4/mbm$), given in factional coordinates for the lattice constants, $a = b$ = 5.791 Å and c = 8.349 Å. All the atomic positions and lattice constants are simultaneously optimized by GGA-PBE. Ti atom displaces by 0.14 Å from its original high-symmetric position.

Atom	Site	x	y	z
Ba	$2b$	0.5000	0.5000	0.5000
Ti	$4g$	0.7326	0.2674	0.0000
Sb	$4f$	0.0000	0.5000	0.2460
O	$2a$	0.0000	0.0000	0.0000

TABLE IV. Internal atomic positions of $BaTi_2Bi_2O$ superlattice structure ($\sqrt{2}\times\sqrt{2}\times 1$, No.127 $P4/mbm$), given in factional coordinates for the lattice constants, $a = b$ = 5.808 Å and c = 8.687 Å. All the atomic positions and lattice constants are simultaneously optimized by GGA-PBE. Ti atom displaces by 0.16 Å from its original high-symmetric position.

Atom	Site	x	y	z
Ba	$2b$	0.5000	0.5000	0.5000
Ti	$4g$	0.7309	0.2691	0.0000
Bi	$4f$	0.0000	0.5000	0.2489
O	$2a$	0.0000	0.0000	0.0000

TABLE V. Internal atomic positions of $BaTi_2As_2O$ superlattice structure ($2 \times 2 \times 1$, No.55 *Pbam*), given in factional coordinates for the lattice constants, a = 8.122 Å, b = 8.108 Å, and c = 7.401 Å. All the atomic positions and lattice constants are simultaneously optimized by GGA-PBE. The magnitude of distortions in terms of the orthorhombicity is evaluated as, $\eta = 2 \times (a - b)/(a + b) = 0.171\%$, being coincidence with the experimental value from neutron diffraction, [6] $\eta = 0.22\%$.

Atom	Site	x	y	z
Ba	$2a$	0.5000	0.5000	0.5000
Ba	$2b$	0.0000	0.5000	0.5000
Ti	$4g$	0.2500	0.5034	0.0000
Ti	$4g$	0.0080	0.2500	0.0000
As	$8i$	0.2519	0.2509	0.2422
O	$2a$	0.0000	0.5000	0.0000
O	$2c$	0.5000	0.0000	0.0000

TABLE VI. T_c obtained by Allen-Dynes formula for $BaTi_2Pn_2O$ (Pn = As, Sb, and Bi)

	Present calculations			Previous calculations			Experiments
Compounds (structures)	λ	ω_{ln}	T_c	λ	ω_{ln}	T_c	T_c
$BaTi_2As_2O$ ($1 \times 1 \times 1$)	1.37	66.56 K	6.93 K	-	-	-	- [1, 7]
$BaTi_2Sb_2O$ ($1 \times 1 \times 1$)	0.62	90.44 K	2.23 K	1.28 [8]	93.52 K [8]	9.0 K [8]	1.2 K [3]
$BaTi_2Bi_2O$ ($1 \times 1 \times 1$)	0.84	74.99 K	3.89 K	-	-	-	4.6 K [4]
$BaTi_2As_2O$ ($1 \times 2 \times 1$)	0.92	137.10 K	8.31 K	-	-	-	- [1, 7]
$BaTi_2Sb_2O$ ($\sqrt{2} \times \sqrt{2} \times 1$)	0.52	165.91 K	2.30 K	0.55 [8]	110 K [8]	2.7 K [8]	1.2 K [3]
$BaTi_2Bi_2O$ ($\sqrt{2} \times \sqrt{2} \times 1$)	0.56	134.39 K	2.45 K	-	-	-	4.6 K [4]

SUPPLEMENTARY NOTE 1

Our optimized geometry parameters for undistorted structures are given in Table I, compared with experimental values. The optimizations were performed under a fixed symmetry, $P4/mmm$, to relax both lattice parameters, a and c, and internal coordinations within a primitive cell. For a and the interanal coordinations, our results are in good agreements with experiments, while those for c are slightly longer than the experimental values. This trend is also reported in previous calculations by Suetin *et al.* [2, 5]. This is due to our choice of GGA-PBE, which is known to overestimate lattice parameters in general [9].

SUPPLEMENTARY NOTE 2

From phonon pDOS (partical DOS), we can identify which vibration modes lead to the instability toward the superlattice. We got pDOS with using QHA module which is implemented in Quantum espresso. [10] For undistorted Pn=Sb and Bi, it is found from Figs.1 and 2 that the negative (imaginary) frequencies mainly come from Ti 'in-plane' (within xy plane) vibrations. This is consistent with the previous calculation by Subedi [8] for Pn=Sb. It is found from Fig. 3 that the negative frequencies of undistorted Pn=As comes from Ti and As 'in-plane' vibrations. Finally, we concluded that only 'in-plane' vibrations contribute to the negative frequencies for all the compound.

SUPPLEMENTARY NOTE 3

To estimate T_c, we used Allen-Dynes formula [11, 12] implemented in Quantum Espresso, [10]

$$T_c = \frac{\omega_{\ln}}{1.2} \exp\left[\frac{-1.04\,(1+\lambda)}{\lambda - \mu^*\,(1+0.62\lambda)}\right], \tag{1}$$

where

$$\lambda = 2\int d\omega \frac{\alpha^2 F(\omega)}{\omega} \tag{2}$$

denotes the frequency-averaged electron-phonon coupling constant, and

$$\omega_{\ln} = \exp\left[\frac{2}{\lambda}\int d\omega\, \alpha^2 F(\omega)\,\frac{\ln\omega}{\omega}\right] \tag{3}$$

denotes logarithm-averaged phonon frequency. The constant, μ^*, describes the effective Coulomb interaction, being chosen 0.1 empirically. Eliashberg function [13] is given as

$$\alpha^2 F(\omega) = \frac{1}{2\pi N(\varepsilon_F)} \sum_{q,\nu} \delta\left(\omega - \omega_{q,\nu}\right) \frac{\gamma_{q,\nu}}{\hbar\omega_{q,\nu}}, \tag{4}$$

where $N(\varepsilon_F)$, $\omega_{q,\nu}$, and $\gamma_{q,\nu}$ denote the density of state at Fermi-level, phonon frequency, and relaxation constant for a mode (q,ν), respectively.

Following the above equation, T_c are estimated for undistorted and superlattice structures Pn =As, Sb and Bi cases. Eliashberg functions are shown in Fig. 4 (undistorted structures) and in Fig. 5 (superlattice structures). The parameters appearing in the formula are also tabulated in the table VI. Our estimated values, T_c = 2.30 (2.45) K for superlattice structures of Pn = Sb (Bi) which show no imaginary frequency, are consistent with experimental values T_c = 1.2 (4.6) K for Pn = Sb (Bi). This evaluation, however, assumes a simple BCS-type mechanism, which might be debatable for $BaTi_2Bi_2O$ as mentioned in the main article. As for Pn = As, T_c were estimated only for the unstable structures which show imaginary frequencies. Therefore, the estimated value T_c = 6.93 K ($1 \times 1 \times 1$), 8.31 K ($1 \times 2 \times 1$) are not compatible with the experimental fact that $BaTi_2As_2O$ does not show any superconductivity. [1, 7]

SUPPLEMENTARY NOTE 4

In general, we can predict superlattice structures by analyzing dynamical matrices. Once we identify the symmetries, we can perform geometry optimizations for the superlattice under the identified symmetries and get relaxed geometry of the superlattice

structures. We found that the lattice instabilities in *Pn* = Sb and Bi induce structural transition from *P*4/*mmm* (No.123) to *P*4/*mbm* (No.127) by analyzing dynamical matrices. The results of geometry optimization under the identified symmetry (*P*4/*mbm*) are shown in Tables III and IV. On the other hand, we found that the lattice instabilities in *Pn* = As induce structural transition from *P*4/*mmm* (No.123) to *Pbmm* (No.51), which is different from *Pn*=Sb and Bi. The results of geometry optimization under the identified symmetry (*Pbmm*) are summarized in Table II. As mentioned in the main article, the superlattice structure of *Pn* = As $(1 \times 2 \times 1)$ still shows imaginary frequencies. We, therefore, further analyzed dynamical matrices and found the structural transition from *Pbmm* (No.51) to *Pbam* (No.55). The results of geometry optimization under the identified symmetry (*Pbam*) are summarized in Table V. The results in Tables II-V are compared with experiments and discussed in the main article.

[1] Wang, X. F. *et al.* Structure and physical properties for a new layered pnictide-oxide: $BaTi_2As_2O$. *Journal of Physics: Condensed Matter* **22**, 075702 (2010).

[2] Suetin, D. & Ivanovskii, A. Structural, electronic properties, and chemical bonding in quaternary layered titanium pnictide-oxides $Na_2Ti_2Pn_2O$ and $BaTi_2Pn_2O$ (*pn* = As, Sb) from FLAPW–GGA calculations. *Journal of Alloys and Compounds* **564**, 117–124 (2013).

[3] Yajima, T. *et al.* Superconductivity in $BaTi_2Sb_2O$ with a d^1 square lattice. *Journal of the Physical Society of Japan* **81**, 103706 (2012).

[4] Yajima, T. *et al.* Synthesis and Physical Properties of the New Oxybismuthides $BaTi_2Bi_2O$ and $(SrF)_2Ti_2Bi_2O$ with a d^1 Square Net. *Journal of the Physical Society of Japan* **82**, 013703 (2013).

[5] Suetin, D. & Ivanovskii, A. Electronic properties and fermi surface for new Fe-free layered pnictide-oxide superconductor $BaTi_2Bi_2O$ from first principles. *JETP Letters* **97**, 220–225 (2013).

[6] Frandsen, B. A. *et al.* Intra-unit-cell nematic charge order in the titanium-oxypnictide family of superconductors. *Nat. Commun.* **5**, 5761 (2014).

[7] Yajima, T. *et al.* Two Superconducting Phases in the Isovalent Solid Solutions $BaTi_2Pn_2O$ (*Pn* = As, Sb, and Bi). *Journal of the Physical Society of Japan* **82**, 033705 (2013).

[8] Subedi, A. Electron-phonon superconductivity and charge density wave instability in the layered titanium-based pnictide $BaTi_2Sb_2O$. *Phys. Rev. B* **87**, 054506 (2013).

[9] Ouma, C. N. M., Mapelu, M. Z., Makau, N. W., Amolo, G. O. & Maezono, R. Quantum monte carlo study of pressure-induced $b3-b1$ phase transition in GaAs. *Phys. Rev. B* **86**, 104115 (2012).

[10] Giannozzi, P. *et al.* QUANTUM ESPRESSO: a modular and open-source software project for quantum simulations of materials. *Journal of Physics: Condensed Matter* **21**, 395502 (2009).

[11] Bardeen, J., Cooper, L. N. & Schrieffer, J. R. Theory of superconductivity. *Phys. Rev.* **108**, 1175 (1957).

[12] Allen, P. B. & Dynes, R. C. Transition temperature of strong-coupled superconductors reanalyzed. *Phys. Rev. B* **12**, 905–922 (1965).

[13] Eliashberg, G. M. Interactions between electrons and lattice vibrations in a superconductor. *Sov. Phys.-JETP* **11**, 696–702 (1960).
